# Gait monitoring for older adults during guided walking: An integrated assistive robot and wearable sensor approach

**DOI:** 10.1017/wtc.2022.23

**Published:** 2022-10-25

**Authors:** Qingya Zhao, Zhuo Chen, Corey D. Landis, Ashley Lytle, Ashwini K. Rao, Damiano Zanotto, Yi Guo

**Affiliations:** 1 Department of Mechanical Engineering, Stevens Institute of Technology, Hoboken, NJ, USA; 2 Department of Electrical and Computer Engineering, Stevens Institute of Technology, Hoboken, NJ, USA; 3 Department of Rehabilitation & Regenerative Medicine (Programs in Physical Therapy G.H. Sergievsky Center), Columbia University, New York, NY, USA; 4 College of Arts and Letters, Stevens Institute of Technology, Hoboken, NJ, USA

**Keywords:** assistive robot, cognitive assessment, dynamic margin of stability, gait analysis, instrumented footwear

## Abstract

An active lifestyle can mitigate physical decline and cognitive impairment in older adults. Regular walking exercises for older individuals result in enhanced balance and reduced risk of falling. In this article, we present a study on gait monitoring for older adults during walking using an integrated system encompassing an assistive robot and wearable sensors. The system fuses data from the robot onboard Red Green Blue plus Depth (RGB-D) sensor with inertial and pressure sensors embedded in shoe insoles, and estimates spatiotemporal gait parameters and dynamic margin of stability in real-time. Data collected with 24 participants at a community center reveal associations between gait parameters, physical performance (evaluated with the Short Physical Performance Battery), and cognitive ability (measured with the Montreal Cognitive Assessment). The results validate the feasibility of using such a portable system in out-of-the-lab conditions and will be helpful for designing future technology-enhanced exercise interventions to improve balance, mobility, and strength and potentially reduce falls in older adults.

## 1. Introduction

An active lifestyle can mitigate physical and cognitive decline in older adults, and prolong functional independence (Hirvensalo et al., 2000; Ahlskog et al., 2011). Walking is a preferred and the most accessible exercise modality among older adults (Morris and Hardman, 1997). Regular walking exercises may improve balance, increase muscle strength, and reduce the risk of falls. Individual or group walking programs are routinely offered at senior centers, either indoor or outdoor (Eyler et al., 2003), but the increasing shortage of trained caregivers due to population aging and increased life expectancy is posing a serious threat to the sustainability of such initiatives in the future. Group activities allow a single trainer to supervise multiple seniors simultaneously, but make it difficult for caregivers to track individual progress and tailor exercise goals to a person’s functional level. Self-administered walking programs are suitable for seniors with moderate impairments, but adherence to the protocols relies on trainees’ *intrinsic motivation* factors, which are difficult to control (Osoba et al., 2019).

Because alterations in walking patterns (i.e., gait speed, gait variability) may be markers of frailty (Montero-Odasso et al., 2005), precursors of fall (Hausdorff et al., 2001), and indicators of neurological or musculoskeletal disorders (Maddox, 2013), gait assessments are often included within health screening for older adults (Winter, 1991). Prior work has shown that analysis of gait under complex conditions, for instance, while performing a secondary cognitive task, can detect important markers of function and future risk for falls (Porciuncula et al., 2016; Osoba et al., 2019). *Traditional* gait analysis relies on clinical observation and timed mobility tests (Steffen et al., 2002) that have moderate discriminatory power (Gates et al., 2008). *Quantitative* gait analysis may provide superior diagnostic power than traditional tests (Verghese et al., 2009), but requires dedicated laboratory space and costly equipment (electronic walkways, motion capture systems, force plates) that most senior centers cannot afford. *Lack of mobility* and *limited workspace* represent two additional drawbacks of these systems, since they constrain the maximum number of consecutive footfalls that can be measured in a given time interval, which negatively affects the reliability of the estimated gait variability (Hollman et al., 2010). Robotics and wearable technology can be leveraged to measure gait parameters over any distance, and administer personalized exercises to community-dwelling older adults. To this aim, researchers have proposed depth imaging sensors, *wearable sensors*, and *assistive robots* (Szymański et al., 2014).

A comprehensive review of the applications of stationary depth imaging sensors in elderly care, including movement analysis and balance training, can be found in Webster and Celik (2014). RGB-D cameras (Stone and Skubic, 2011; Gabel et al., 2012; Clark et al., 2013) and laser range sensors (LRS) (Pallejà et al., 2009; Yorozu et al., 2014) can estimate a basic set of gait parameters, but they share some of the drawbacks of optical motion capture systems, for example, constrained workspaces and occlusions. Stationary cameras also require costly modifications to an individual’s home, and their acceptance is hampered by users’ privacy concerns. In camera-based balance trainers, the user’s movements are continuously compared to a database of template movements, and the user is provided with real-time feedback on his/her performance (Lange et al., 2011; Kayama et al., 2013; Lin et al., 2013).

Wearable systems consist of a network of sensors, a smartphone to log and relay data to a remote unit, and a data analysis unit that converts these signals into clinically relevant information (Patel et al., 2012). Wearable sensors have been used for gait assessments and to administer game-like balance training exercises in older adults (De Morais and Wickström, 2011; Rao, 2019). Among the wearable systems for gait assessments, in-shoe devices are promising since they allow for minimally obtrusive, ubiquitous measurements (Hegde et al., 2016; Zanotto et al., 2017). Yet, compared to laboratory equipment, they can measure a limited set of gait parameters, which are typically restricted to the sagittal plane. While in-shoe devices can reliably estimate temporal gait parameters, they are less accurate than laboratory equipment in measuring spatial parameters (Mariani et al., 2010); Rampp et al., 2015; Minto et al., 2016; Zhang et al., 2022).

The use of mobile robots to administer exercises has also been proposed in recent years. Compared with virtual trainers, robots’ physical embodiment is thought to increase seniors’ engagement and intrinsic motivation – both critical factors for the success of rehabilitation interventions – as robots may exhibit human-like social behaviors (Bainbridge et al., 2011; Fasola and Mataric, 2012). To date, most studies have focused on chair aerobics (Fasola and Mataric, 2010; Gorer et al., 2017), while limited research has explored the use of mobile robots as tools for gait analysis (Yorozu and Takahashi, 2015) or walking exercises (Piezzo et al.,^2017^) for older adults. In these applications, limited workspace and obstruction-related issues typical of depth image sensors are mitigated by leveraging the robot’s *mobility* (Leica et al., 2015; Piezzo and Suzuki, 2017).

While wearable sensors and mobile robots equipped with onboard depth image sensors may meet the mobility requirements of a portable system capable of administering *ubiquitous* and *autonomous* gait assessments and walking exercises to older adults, the potential behind their combined use has been largely overlooked thus far (Cifuentes et al., 2014; Moschetti et al., 2019). In this article, we present an integrated system consisting of a mobile robot and in-shoe sensors, where the mobile robot guides the older adult to walk on a designated track during overground walking exercises. Together with the in-shoe sensors that the older adults wear, the robot autonomously measures spatiotemporal gait parameters in real time, and estimates the dynamic margin of stability (MoS) for potential assessment of the fall risk. We validate the system with older adults at a community center. Performance evaluations of the guided robot control are reported with satisfactory results. Accuracy of the autonomous gait parameters estimation is quantified using a validated electronic walkway, and the results show comparable or better performance than existing methods. Associations between gait metrics, physical performance, and cognitive ability are analyzed, revealing larger increases in gait variability and more pronounced adaptations toward conservative gait strategies in older adults with higher levels of cognitive impairment performing a secondary cognitive task. Survey results that measure participants’ attitudes toward technology and our integrated system are reported with summarized data.

While our recent work (Zhang et al., 2020; Chen et al., 2022) focused on spatiotemporal gait analysis and MoS estimation, and validated these methods with healthy individuals under controlled laboratory conditions, this article focuses on validation of the system with older adults at a community center in guided walking exercises with a clinically-oriented protocol. New contributions include the following: (1) a design of mobile robot motion planning and distance-keeping control to guide older adults in walking exercises; (2) an evaluation of the system’s accuracy on gait monitoring during walking exercises with older adults in a community center; (3) an investigation of how the Montreal Cognitive Assessment (MoCA) score, a clinical measure of cognitive function, is independently associated with changes in gait and balance metrics captured by the integrated system during dual-task walking in older adults; (4) an analysis of older adults’ attitude toward the proposed technology.

The remainder of this article is organized as follows. Section 2 presents our integrated robot and wearable sensor system and the experimental protocols. Section 3 presents the robot subsystem design and its performance validation, where the robot maps the environment, localizes itself, and autonomously controls its motion for path tracking and distance keeping with the human subject. The autonomous gait parameter monitoring and MoS estimation are described in Section 4 with performance validation. The association between physical performance, cognitive ability, and gait parameters is presented in Section 5. The participant attitude survey results are presented in Section 6. Study limitations are discussed in Section 7, together with directions of future work. Finally, the article is concluded in Section 8 with brief remarks.

## 2. System and experimental protocol

### 2.1. Integrated robot and wearable sensor system

**Figure 1.**
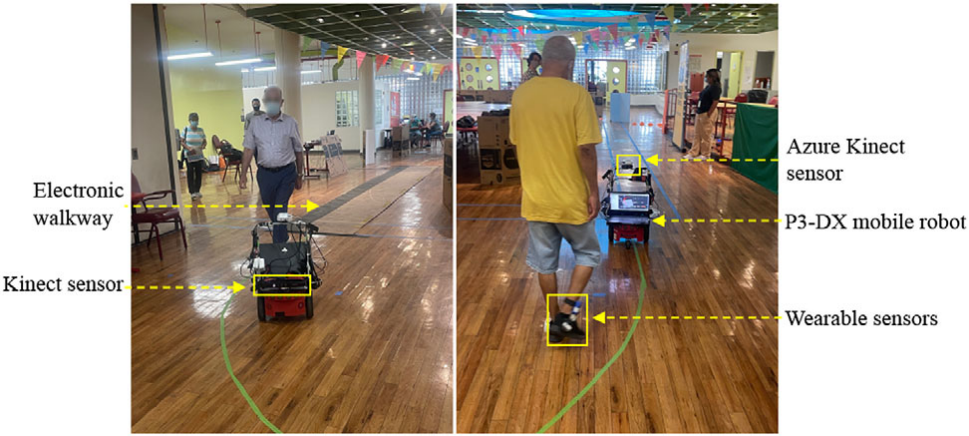
The system consists of a P3-DX mobile robot and an instrumented footwear subsystem. The Azure Kinect sensor (shown in the right picture) is used for gait monitoring. The Kinect sensor (shown in the left picture) is used for robot mapping and localization. A validated electronic walkway is used as the reference system to validate the system’s accuracy in measuring gait parameters.

Our system consists of a wheeled mobile robot and an instrumented footwear subsystem. The mobile robot is a customized P3-DX differential drive robot equipped with a laptop computer (Intel Core i7-9750H CPU, Nvidia RTX 2060 GPU) that works as the onboard computing device, a backward-facing Azure Kinect sensor for gait monitoring, and a forward-facing Kinect v1 sensor for mapping and localization. The instrumented footwear subsystem (Zhang et al., 2017, 2020) consists of a pair of insoles, each featuring eight piezoresistive cells (from IEE S.A., Luxemburg), underneath the left and right calcaneus, the lateral arch, the head of the first, third and fifth metatarsals, the hallux, and the toes, respectively. An inertial measurement unit (IMU, Yost Labs Inc., Portsmouth, OH) is embedded in each insole under the medial arch. Together with the insole, a Li-Po-battery-powered logic unit is clipped laterally on the shoe of the subject, which is composed of a 32-bit ARM Cortex-M4 microcontroller and a Wi-Fi module for the purpose of streaming data from the IMU and pressure sensors to the robot. The robot and the logic units of the instrumented footwear communicate in a local area network through a wireless router. The laptop computer on the robot controls the robot’s motion and processes the data from both the robot and instrumented footwear subsystems. Programs for robot motion control, data acquisition, and gait analysis run as Robot Operating System (ROS) nodes in Ubuntu 18.04 with ROS Melodic. Figure 1 shows the integrated robot and wearable sensor system leading a study participant in a walking exercise.

### 2.2. Experimental protocol

We recruited 24 participants from the Center for Active Older Adults in the Sunnyside Community Services (Queens, NY). The center offers meals, activities, and exercise classes for older adults. Participants were recruited into the study if they were (1) between the ages of 65–85 years, (2) regularly attended the Center for Active Older Adults, (3) Able to walk a distance of 50 m independently, (4) Willing and able to follow the study protocol, and (5) Able to understand English or Spanish. We excluded participants if (1) They had an acute medical illness 30 days before study participation, (2) had a history of cardiopulmonary, neurological or musculoskeletal disorder that affected their ability to walk, (3) Had a history of heart disease or uncontrolled blood pressure, (4) loss of sensation in the lower limbs, and (5) History of seizure disorder. Study procedures were approved by the Institutional Review Board (IRB) at Columbia University Medical Center (Protocol #: AAAS0003) and the IRB at Stevens Institute of Technology (Protocol #: 2019–014). The research team and center staff screened potential participants for eligibility. All participants were explained the study purpose and procedures, and provided written informed consent before participating in the study. Following consent, we recorded demographic and anthropometric data, administered the cognitive test (MoCA) and the Short Physical Performance Battery (SPPB).

#### 2.2.1. Assessments

##### 2.2.1.1. Demographic and anthropometric information

We recorded the following information from each participant: date of birth, sex, race, ethnicity, handedness, highest level of education, and history of injury to the lower limbs in the past 6 months. We also recorded anthropometric data such as height, weight, leg length, and shoe size. The anthropometric data were used for calculation of gait data.

##### 2.2.1.2. Montreal Cognitive Assessment

In order to screen for cognitive deficits, a trained researcher administered the MoCA. The MoCA is a quick screening tool that has been extensively tested in older adults (Luis et al., 2009; Dale et al., 2018). In addition, participants performed a serial three counting backward task. Participants were given a three-digit number and were asked to count backward by three for a period of 1-min. We recorded the number of digits counted and errors. The serial three task was used as a baseline to compare with the performance of the same task while walking.

##### 2.2.1.3. Short Physical Performance Battery

The SPPB is a set of three tests that assess lower extremity strength, balance, and mobility in older adults. Two trained researchers administered the SPPB. To assess functional strength, participants performed a timed five times sit-to-stand task. We assessed balance by asking participants to stand for 10 s with their feet in three different positions (together side-by-side, semi-tandem with one foot slightly in front of the other, and tandem with one foot directly in front of the other with the heel of the front foot touching the toe of the rear foot). We recorded the time for each of the three tasks. To assess mobility, participants completed two trials of timed 4-m walk. Administering the SPPB took approximately 10 min. The SPPB has been extensively tested for reliability and validity in older individuals (Guralnik et al., 1994). A summary of the study participants’ demographic data, anthropometric data, and assessment scores is reported in Table 1.

**Table 1.** Demographic information, MoCA scores, and SPPB scores

	*N* = 24
Age, mean (SD)	75.8 (5.4)
Sex, *n* ( 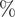 )	
Male	8 (33.3 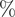 )
Female	16 (66.7 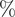 )
Height [cm], mean (SD)	160.7 (7.0)
Weight [kg], mean (SD)	67.5 (12.5)
MoCA [0–30], mean (SD)	21.5 (3.6)
Cognitive impairment ( 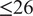 ) (Damian et al., 2011)	100 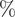
SPPB [0–12], mean (SD)	8.3 (1.5)
Risk for disability ( 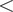 10) (Guralnik et al., 1994)	75 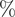

Abbreviations: MoCA, Montreal cognitive assessment; SPPB, short physical performance battery.

##### 2.2.1.4. Normal and dual-task walking

**Figure 2.**
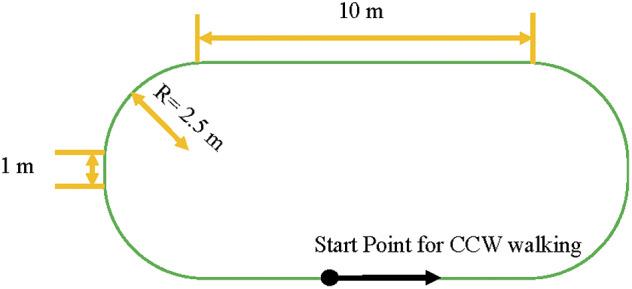
Dimensions of the human path marked on the floor in the community center where all experiments were conducted.

Following these assessments, participants were provided with instrumented insoles of appropriate size and were oriented to the mobile robot. The experiment was conducted in the common area at the community center. An oval path, approximately 38-m long, was marked on the floor with adhesive tape to serve as the nominal path for all the walking trials (Figure 2). First, each participant walked two laps along the marked oval path, at their preferred speed to familiarize with the integrated system (familiarization trial, FS). Following the FS, each participant completed two walking trials, normal walking trial (N), and dual-task walking trial (D), each consisting of four laps along the same oval path, while their gait was tracked by the integrated robot/insole system. The normal walking trial required subjects to walk at their preferred speed. The dual-task walking trial required participants to walk at their preferred speed while counting backward by three, starting from a random three-digit number. The trial sequence (N, D) as well as the direction of the walking task (clockwise, counterclockwise) were balanced across the study participants using a Latin square design.

#### 2.2.2. COVID-19 safety protocol

Data were collected between July 13, 2021 and August 26, 2021, during six separate visits to the community center. As data collection took place during the COVID-19 pandemic, safety protocols were in place to protect the participants, staff, and the research team. The NYC Department for the Aging required all persons to present either proof of vaccination or a negative PCR test to enter the center. In addition, an indoor mask mandate was in place during testing. At the time of data collection, the average COVID-19 infection rate in the NYC area was 408 cases on July 13 (first day of data collection) and 1,899 cases on August 26 (last day of data collection; The New York Times, 2022).

## 3. Mobile robot localization and control design

During the “Normal and Dual-task Walking” as described in the previous section, the robot leads the participant to walk on the oval path and monitors the participant’s gait. To perform this task, the mobile robot is programmed to autonomously map the environment, track the participant’s body joints using its onboard RGB-D sensor, and maintain a certain distance from the participant while he/she walks on the oval path marked on the ground. In this section, we describe the autonomous mapping and localization method, robot motion planning, and distance-keeping controller design. Validation data and performance evaluation are presented at the end of this section.

### 3.1. Robot mapping and localization

**Figure 3.**
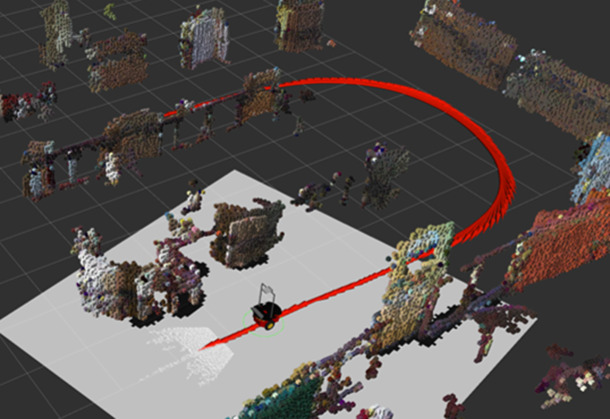
Map generated by RTAB-Map using visual-SLAM, and the path (red-colored curve) planned by the robot.

For the robot to guide the participant to walk on the marked oval path, the robot first needed to map the environment and localize itself in the map during the guided walking trial. RTAB-Map (Real-Time Appearance-Based Mapping), an open-source library (Labbé and Michaud, 2019), is used for visual Simultaneous Localization and Mapping (SLAM), which fuses the robot’s wheel odometry with the RGB-D data from the forward-facing Kinect v1 (i.e., Kinect for XBox 360) sensor on the robot. To map the environment, we teleoperated the robot using a wireless keyboard and drove the robot along the marked track several times in both clockwise and counter-clockwise directions, which allowed feature building and loop closure in the SLAM process. The generated map is visualized in RViz (a 3D visualization tool for ROS applications) in Figure 3 with obstacles rendered by colored cubes.

### 3.2. Robot motion planning and distance-keeping control

**Figure 4.**
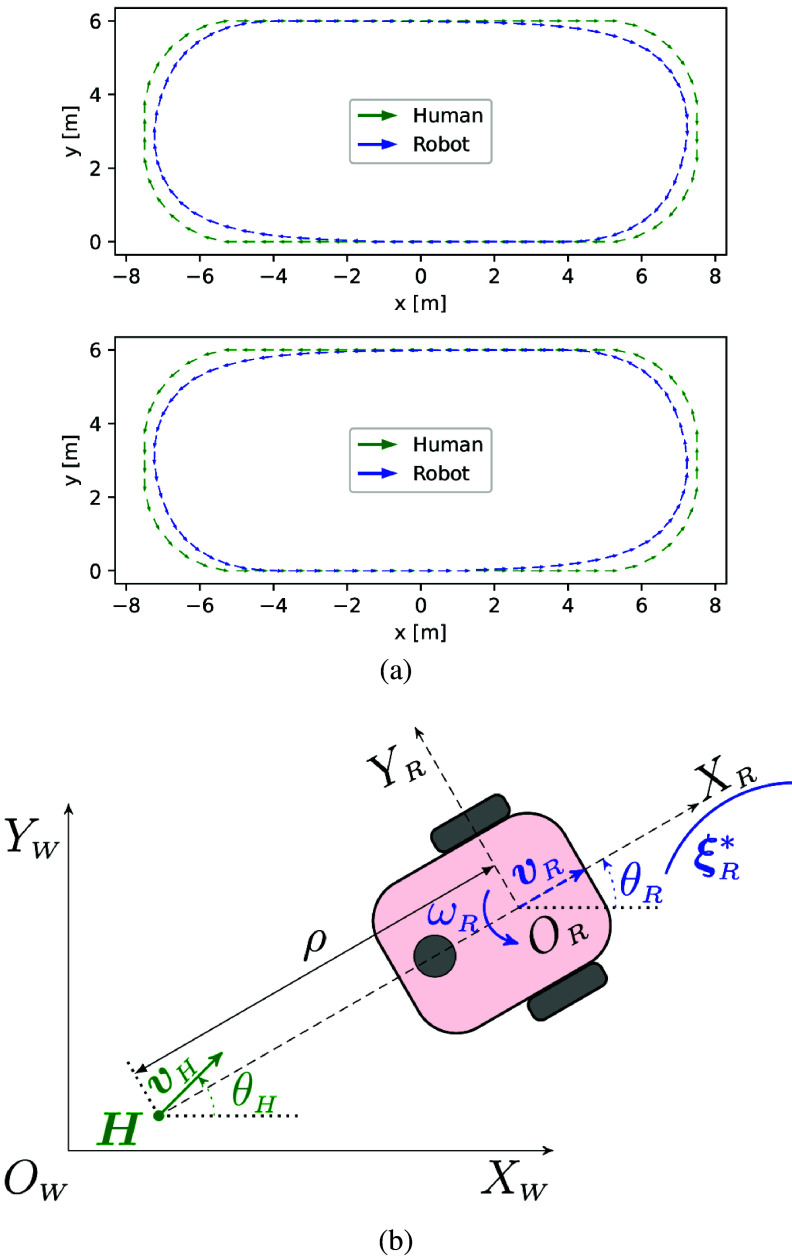
(a) The planned paths for the human (in green) and the robot (in blue). (b) The relative position of the human (denoted by H) and the differential drive robot during guided walking. The 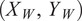 axes represent the world (or global) coordinates, and the 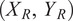 axes represent the robot (or local) coordinates.

To plan the robot motion trajectory, we design a global robot path as shown in Figure 4a, so that the human can always be in the field of view (FOV) of the robot during walking. Let 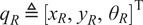 be the robot configuration vector. The kinematic model of the P3-DX differential drive robot can be written as follows:
(1)

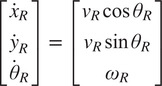

where 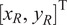 is the midpoint of the two wheels, 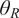 denotes the heading of the robot, and the control vector 
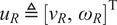
 includes the linear velocity 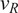 and the angular velocity 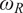 .

To generate the robot control input 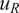 , we utilize a local motion planner, Dynamic Window Approach (DWA)^1^ (Fox et al., 1997). This method searches a set of trajectories, each of which consists of a sequence of achievable velocities in the planning horizon, for the robot to get from the current pose to the desired pose. Let the desired distance between the human and the robot be 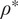 , which is chosen to be 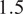 m in our experiments. To maintain this distance, we design a proportional-integral (PI) controller:
(2)

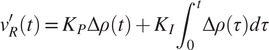

where 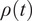 is the measured distance between the robot and the human, and 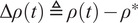 is the measured distance error. The control parameters were chosen as 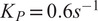 and 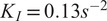 . Directly using the linear velocity 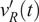 in 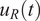 would change the global path planned previously; for the robot to track the planned path 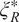 , we scale the control input 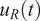 as
(3)

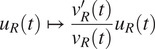

This scaling does not change the curvature of the tracked path and hence preserves the planned global path. The robot keeps the desired distance 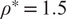 m by changing its speed (i.e., the linear velocity 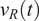 ) according to the human’s actual walking speed.

### 3.3. Performance of the robot controller

**Figure 5.**
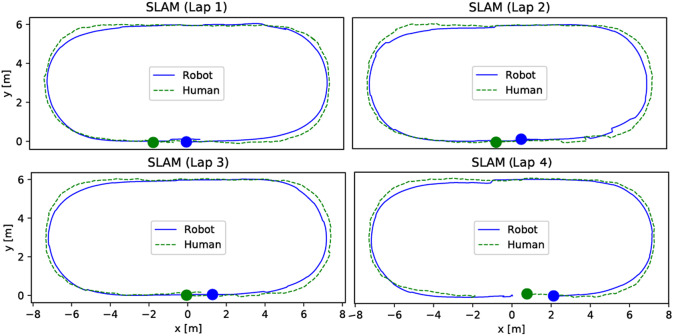
Robot and human paths during four laps of a representative walking trial. The solid dots denote the start positions of the robot (in blue) and the study participant (in green). In this trial, the study participant walked in the counter-clockwise (CCW) direction.

Figure 5 shows the robot and participant paths of a representative four-lap walking trial recorded by the robot computer using the pose estimate provided by the onboard SLAM algorithm. As shown in the plots, the robot follows the planned trajectories with satisfactory performance.

**Figure 6.**
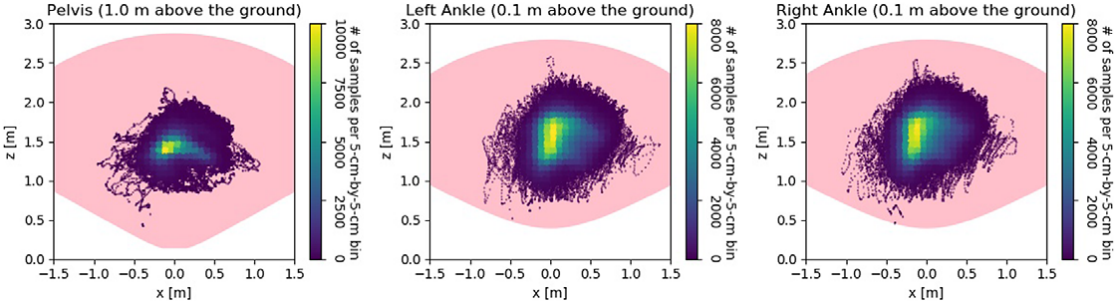
Top view of the sample distribution of the joint position measurement (relative to the Kinect depth FOV), including pelvis (left), left ankle (middle), and right ankle (right), using 22 subjects’ data collected in the study. The pink region is the intersection of the depth FOV, and the height of the horizontal plane is indicated on the top of each subfigure.

One key design goal of the path planning algorithm is to keep the following participant in the center of the FOV of the Azure Kinect sensor, so that the robot can track the human joint movement in the gait monitoring task (to be discussed in Section 4). Figure 6 shows the collected samples of the Azure Kinect sensor measurement of the joint positions from 22 participants in the study. A total of 24 older adults participated in the walking tests, but due to technical issues (discussed in Section 7), robot control data for two participants were not available to use. The Kinect sensor depth FOV takes the shape of a truncated cone with the near clipping plane at 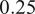 m from the optical center, the far clipping plane at 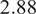 m, and the apex angle being 120°. It can be seen that the Kinect sensor measurement of the human joints is mostly centered in the FOV.

**Figure 7.**
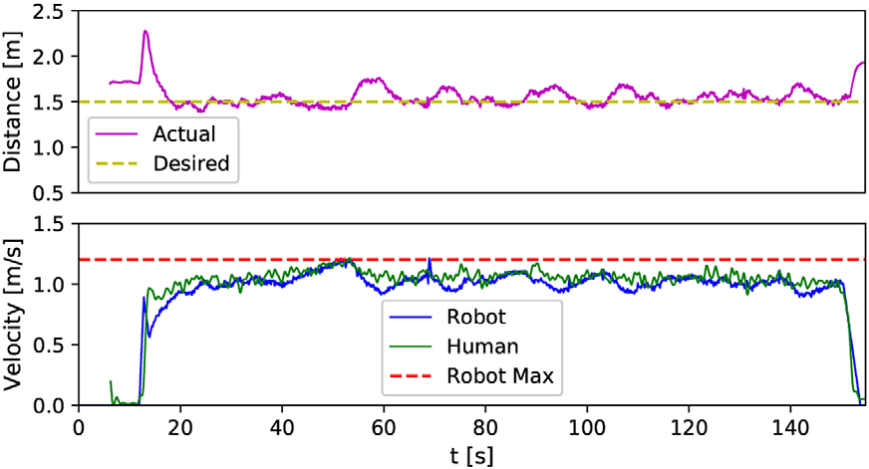
Robot distance-keeping performance in a representative four-laps walking trial. Time histories of the actual distance between the robot and the following participant, measured by the robot onboard sensors (Top). Time histories of the robot’s and the participant’s speeds, measured by the robot onboard sensors (Bottom).

Figure 7 shows the distance-keeping performance of the robot controller. As shown in Figure 7(top) the human–robot distance is maintained around the desired value of 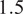 m. Additionally, Figure 7(bottom) indicates that the robot can match the participant’s walking speed. The fluctuation of the trajectories is caused by the measurement noise and the feedback nature of the controller (2) that uses the measured human–robot distance to control the robot.

**Figure 8.**
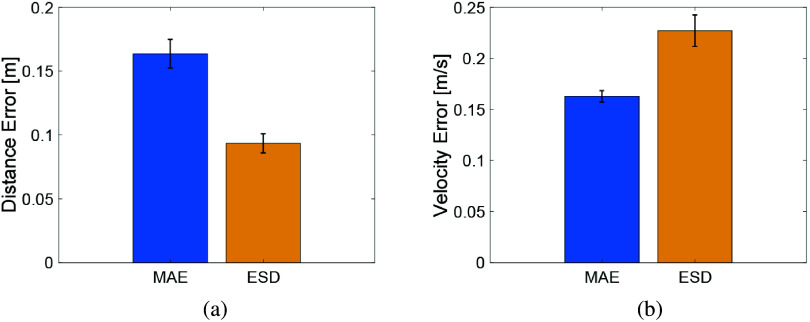
Bar plots of MAE and ESD for 22 participants: (a) human–robot distance error, and (b) human–robot velocity error.

We calculated the mean absolute error (MAE) and the error standard deviation (ESD) for both the distance and the velocity errors between the robot and the human, across all 22 participants, as shown in Figure 8. We excluded the initial 15 s and the last 5 s of each walking trial, as those are transient periods for the controller to stabilize. The distance error is lower than 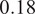 m and the velocity error is below 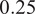 m/s. Thus, the robot can guide the participants to walk on the pre-designated path, and can maintain the desired distance from the participant during the walking exercise.

## 4. Autonomous gait monitoring and MoS estimation

During the robot-guided walking, the backward-facing Azure Kinect (RGB-D sensor) located on the robot tracks the pelvis and foot poses of the participant following the robot. extended Kalman filter (EFK)-based methods were developed to fuse the data from the robot RGB-D sensor and the in-shoe pressure sensors and IMUs and estimate spatiotemporal gait parameters and the MoS. We briefly describe the methods in the next two subsections.

### 4.1. Estimation of spatiotemporal gait parameters

Temporal gait parameters were extracted using insole-embedded force-sensitive resistors (FSRs). Heel strike (HS) and toe-off (TO) events were detected when the sum of the FSR signals crossed an empirically-determined threshold. Stride time (ST) was defined as the time interval between two consecutive HS of the same foot. Swing time was defined as the time interval between a TO event and the following HS of the same foot. Swing percent (SwP) was computed as swing time divided by the ST of the corresponding stride. Spatial gait parameters were extracted by fusing data from the in-shoe IMUs and the robot onboard RGB-D camera. The angular velocity and acceleration obtained from the IMUs, and the poses of the IMUs obtained from the robot onboard camera were fed in the EKF to estimate the foot poses. Foot-flat (FF) phases are determined as the time intervals during which the normalized acceleration of the IMU was less than a predefined threshold. Stride length (SL) was determined as the distance between two successive IMU locations of the same foot at FF. Stride velocity (SV) was computed as the ratio of SL over ST. A detailed description of this method is presented in Chen et al. (2022).

### 4.2. MoS estimation

MoS was proposed by Hof et al. (2005) as a measure of stability in human movement control. Modeling the human as an inverted pendulum, Hof et al. postulated that the condition for maintaining balance is that the extrapolated center of mass (XCoM) falls inside the Base of Support (BoS). The MoS has been extensively used to analyze dynamic balance in older adults (Watson et al., 2021), whereas limited research has focused on overground walking tasks (Ohtsu et al., 2020; Iwasaki et al., 2021), which are more representative of real-world walking. In estimating MoS, the RGB-D sensor on the robot tracks the participant’s pelvis, transfers the measurement from the sensor frame to the world frame, and projects it to the 2D ground position as the body center of mass (CoM). After estimating CoM and its velocity, XCoM was obtained as the sum of the CoM and a term proportional to the velocity of CoM (Hof et al., 2005). BoS was determined by the estimated foot poses using the convex hull of the set of vertices of the BoS polygon. The MoS was calculated at each timestamp as the signed distance between the BoS and XCoM (positive if XCoM is inside the BoS, and negative otherwise). For each gait cycle, the MoS time series was time-normalized into 100 equally spaced points in the gait phase domain and projected onto the anteroposterior (AP) and mediolateral (ML) axes. Subsequently, the following three scalars were extracted at each gait cycle: 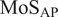 was the mean of the AP projection of the MoS measured over the gait cycle; 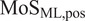 ( 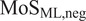 ) was the positive (negative) ML projection of the MoS integrated over the gait cycle. More details on the EFK-based method for MoS estimation can be found in Chen et al. (2022).

### 4.3. Validation of gait monitoring capability and comparison with related works

A total of 2,562 strides were simultaneously collected by the integrated mobile robot and wearable sensor system, and by a validated electronic walkway (a 6-m Zeno Walkway, Protokinetics LLC, Havertown, PA). The electronic walkway, which served as the reference system for validation purposes, was located in the middle of the straight-line section of the oval path shown in Figure 2. A total of 24 older adults participated in the walking tests. Due to technical issues, estimations of spatial gait parameters and MoS were not available for two participants. During the normal walking trials, participants’ SL ranged from 0.85 to 1.42 m (1.12 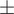 0.14 m, mean 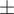 SD), SV ranged from 0.56 to 1.27 m/s (0.98 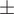 0.20 m/s), step width (SW) ranged from 0.02 to 0.15 m (0.09 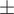 0.03 m), ST ranged from 0.97 to 1.52 s (1.17 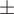 0.14 s), and SwP ranged from 26.35 to 35.60% (35.11 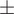 2.79%).

MAE of the spatiotemporal gait parameters were determined by comparing stride-by-stride gait metrics extracted from the integrated system with the corresponding values measured by the reference walkway. Data are reported in Table 2, along with results from recent related studies. In general, gait analysis systems based on IMUs show lower accuracy in estimating spatial gait parameters. This indicates that conventional error reduction techniques such as zero-velocity updates (ZUPT) (Ferrari et al., 2015) and velocity de-drift (Rampp et al., 2014), which are often used in IMU-based devices, cannot fully eliminate accumulated errors in the foot displacements. Renggli et al. (2020) reported higher accuracy than similar IMU-based systems; however, the accuracy of their system was validated using only 60 strides from three subjects. Another drawback of IMU-based devices is the difficulty in estimating the relative position of the feet to determine spatial inter-limb gait parameters. To overcome this issue, Renggli et al. (2020) used the tilting angle at the FF phase and a predefined distance between the feet to estimate SW; however, this method resulted in lower accuracy compared to robot onboard cameras (Piezzo et al., 2017). Robot onboard cameras represent a promising method to capture both inter- and intra-limb spatial gait parameters, but their accuracy relies on the robot’s ability to maintain a predefined distance between the subject and the camera. Cifuentes et al. (2014) reported higher accuracy than other robot and IMU-based systems; however, the accuracy of their prototype was evaluated at low speeds (i.e., <0.4 m/s) and their definition of velocity does not conform to the conventional definition of SV. As described in Section 4.1, in our integrated system in-shoe FSRs were used to obtain temporal gait parameters (ST, SwP), the robot onboard camera and the IMUs were used to estimate spatial gait parameters (SL, SW), and combined data was used to calculate SV. This approach resulted in higher accuracy in terms of spatiotemporal gait parameters compared to the IMU-based system introduced by Rampp et al. (2014) and the robot-based systems described in Jäschke et al. (2018) and Guffanti et al. (2021). A possible explanation is that detecting HS and TO events from IMU acceleration peaks, as done in Rampp et al. (2014), might not be an accurate strategy with older adults, who often show unclear gait events (e.g., shuffling gait). In that same study, the accumulated error in the double integration process might have lowered the accuracy of spatial gait parameters. For the robot-based systems presented in Jäschke et al. (2018) and Guffanti et al. (2021), spatiotemporal gait parameters were estimated by a Kinect sensor based on the position of a person’s ankle, instead of the real foot placement, and this approximation might have contributed to lower the accuracy of their systems.

**Table 2.** Accuracy of the spatial-temporal gait parameters estimated by different systems

References	Subjects	Population	Systems	MAE				
				SL (m)	SV (m/s)	SW (m)	ST (s)	SwP (%)
Zhang et al. (2022)	95 OA	frail	IMU + FSR	0.059	0.048	—	0.017	—
Renggli et al. (2020)	3 A	healthy	IMU	0.029^a^	0.018^a^	0.092^a^	0.024^a^	—
Rampp et al. (2014)	101 OA	geriatric inpatients	IMU	0.063	—	—	0.029	—
Mariani et al. (2010)	10 OA	healthy	IMU	>0.030	>0.030	—	—	—
Foo et al. (2022)	7 A	healthy	Robot	—	—	0.054	0.035	—
Jäschke et al. (2018)	3 A	2 healthy, 1 hip dysplasia	Robot	—	0.029	—	0.014	—
Piezzo et al. (2017)	3 A	healthy	Robot	—	—	0.025^b^	—	—
Bonnet et al. (2015)	1 A	—	Robot	0.022	—	—	—	—
Moschetti et al. (2019)	19 A	healthy	Robot + IMU	0.054	0.067	—	—	—
Cifuentes et al. (2014)	1 A	—	Robot + IMU	—	0.005	—	—	—
Our Work	24 OA	healthy	Robot + IMU + FSR	0.019	0.018	0.048	0.008	3.220

a Estimated based on the reported mean error and standard deviation, assuming a normal distribution.

b Estimated as the ratio between the sum of the reported MAE and the number of the subject.

Abbreviations: A, adults; FSR, force-sensitive resistor; IMU, inertial measurement unit; MAE, mean absolute error; OA, older adults; SL, stride length; ST, stride time; SV, stride velocity; SW, step width; SwP, swing percent.

## 5. Associations between physical performance, cognitive ability, and gait parameters

We explored how the spatiotemporal gait parameters and MoS measured during the N and D trials correlated with MoCA scores and SPPB scores. To this end, Hierarchical linear regression was used to determine if SPPB and MoCA scores were independently associated with three groups of gait parameters: (1) mean and coefficient of variation (CV) of SW, SL, SV, ST, SwP, 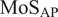 , 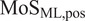 , and 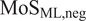 , separately for trials N and D; (2) differences of the mean and CV values of each gait parameter between the two trials (i.e., 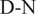 ); (3) ratio of the mean and CV values of each gait parameter between the two trials (i.e., D/N). Differences and ratios of the mean values of gait parameters measured during dual-task walking and natural walking have been used in previous works to identify fallers (Commandeur et al., 2018) and to explore balance strategies (Ohtsu et al., 2020). Because dynamic MoS and spatiotemporal parameters are affected by age and gender (Lee et al., 2021), we included both age and gender as predictors in the base models. The complete models differ from the base models in that they include either SPPB or MoCA as additional predictors. SPSS v28 (IBM Corporation, Armonk, NY) was used to perform all analyses. All models resulting in significant ( 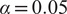 ) associations between SPPB (or MoCA) and one gait parameter are reported in Table 3. SL was positively correlated with SPPB scores, and 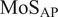 was negatively correlated with SPPB scores. Moreover, the changes in variability of ST and SV between the N and D trials were negatively correlated with MoCA scores, and the D/N ratio of 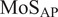 was positively correlated with MoCA scores.

**Table 3.** Multiple regression models

		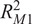	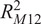	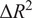	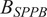	(95%CI)	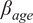	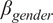	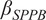
MEAN	SL_N	0.115	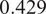 ^b^	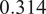 ^b^	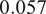 ^b^	(0.020,0.094)	0.009	−0.202	0.609
	SL_D	0.218	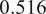 ^b^	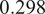 ^b^	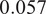 ^b^	(0.022, 0.092)	−0.199	−0.096	0.593
	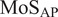 _N	0.034	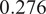 ^a^	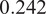 ^a^	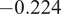 ^a^	(−0.046, −0.002)	−1.116	0.169	−0.521
	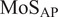 _D	0.095	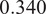 ^a^	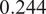 ^a^	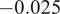 ^a^	(−0.045,-0.005)	−0.083	0.252	−0.537

*Note.* 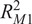 and 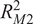 are the coefficients of determination for the base models (age, gender) and for the complete models (age, gender, SPPB or MoCA), respectively. 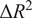 is defined as 
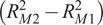
 . Regression coefficients 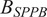 and 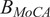 are reported along with their 95% confidence intervals (CI). 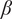 indicates the standardized regression coefficient for each predictor in the complete models. Suffixes 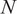 and 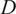 indicate normal and dual-task walking, respectively.

a *p* < .05;

b *p* < .01;

Abbreviations: MoCA, Montreal cognitive assessment; SPPB, short physical performance battery.

The results shown in Table 3 suggest that SPPB and MoCA are associated with distinct gait domains. The values of the standardized coefficients ( 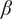 ) indicate that SPPB and MoCA had stronger predictive ability than age and gender in all the significant models. Moreover, consistent with previous research (MacAulay et al., 2015), SPPB scores were positively associated with SL. Knee extensor muscles contribute to SL (Jabbar et al., 2021) and SPPB evaluates strength in these muscles through the five times sit-to-stand component of the assessment (Mentiplay et al., 2020). Additionally, static balance performance, which SPPB evaluates through three standing balance sub-tests, is known to be positively correlated with SL (Lee et al., 2020). Thus, both associations can explain the correlation between SPPB and SL. The negative association between SBBP and 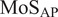 was possibly mediated by SL, since 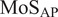 is known to decrease as SL increases (Lencioni et al., 2020). Interestingly, SPPB was not associated with SV, even though one component of the SPPB compound score specifically targets gait speed. One possible explanation is that SPPB determines preferred walking speed by relying on a short (3 or 4 m) walking test, whereas in our tests SV was computed as the average gait speed over a 150-m walking bout. Hence, the estimates of SV were likely affected by fatigue. In our sample, older adults with lower levels of cognitive impairment (i.e., higher MoCA scores) showed smaller increases in gait variability and less-pronounced AP adaptations when performing a secondary cognitive task. Associations between increased stride-to-stride fluctuations in gait parameters and cognitive decline have been consistently reported in the literature (Pieruccini-Faria et al., 2021). Such associations have been linked to shared brain networks for gait control and cognition, which are challenged by dual-task walking (Morris et al., 2016). Furthermore, a smaller ratio of 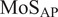 between fast and preferred gait speed is an indicator of conservative gait strategies in older adults at risk of falling (Ohtsu et al., 2020). Similarly, our results on the D 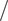 N ratio of 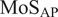 suggest that older adults with higher levels of cognitive impairment tend to show more marked AP adaptations toward conservative gait patterns when performing a secondary cognitive task.

## 6. Subject attitude survey results

**Figure 9.**
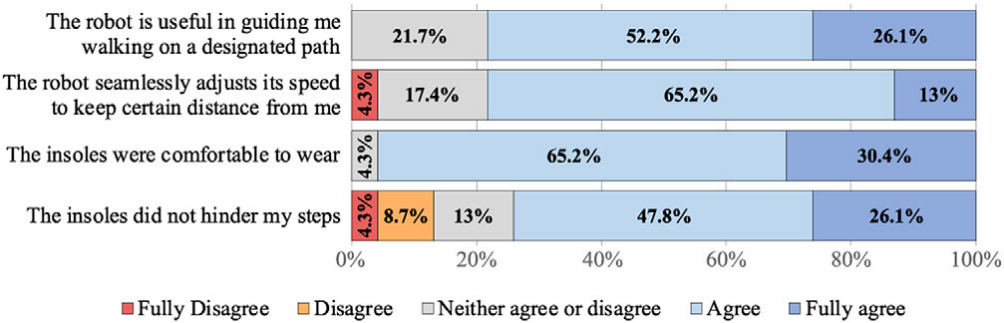
Summary of subject attitude survey.

After engaging in the walking exercise, 23 participants answered questions about the assistive robot and the insoles. One participant did not answer any of these questions. Participants answered two questions about the assistive robot: “The robot is useful in guiding me walking on a designated path” (*M* = 4.04, SD = 0.71) and “The robot seamlessly adjusts its speed to keep a certain distance from me” (*M* = 3.83, SD = 0.83) on a 1 (fully disagree) to 5 (fully agree) scale. Participants also answered two questions about the insoles “The insoles were comfortable to wear” (*M* = 4.26, SD = 0.54) and “The insoles did not hinder my steps” (*M* = 3.83, SD = 1.07) on a 1 (fully disagree) to 5 (fully agree) scale. A distribution of participant responses to each of the four questions is shown in Figure 9. Participant’s attitudes regarding both the assistive robot and insoles were predominately positive, noting that the assistive robot was useful in guiding them on a walking path (78.3% agreed or fully agreed) and was able to adjust speed appropriately (78.2% agreed or fully agreed). Similarly, participants reported that the insoles were comfortable (95.6% agreed or fully agreed) and did not hinder their ability to walk (73.9% agreed or fully agreed).

In addition, participants were asked “It is likely that I’ll use such a robot in my home for guided walking exercises” (*M* = 2.83, SD = 1.53) on a 1 (fully disagree) to 5 (fully agree) scale. A follow-up question asked participants to further explain their answer. Seven participants provided a response to the open-ended question. Sample responses included seeing the utility in having an assistive robot to help at home: “When I think of people who are currently stuck at home because they cannot move around as before, I wish they had the opportunity to have some kind of robot to help them live a better life without an outside help.” In a similar comment, a participant noted that they “would take the robot and use it at home.” Another participant mentioned being interested in using an assistive robot to exercise: “would like to use it as I like everything that is exercise.” Lastly, three participants mentioned a lack of space in their small apartments as being an unknown in personal usage, for example, “My apartment is tiny, it would need to be a small robot in order for me to consider using at home” “I would take the robot and use it at home.” These open-ended responses generally align with the positive attitudes found in the quantitative measures but include some nuance surrounding limitations (e.g., living in a small space) that might hinder personal usage. Given that this study was conducted in New York City, living in a small apartment is common for many. As such, concerns about space issues when using an assistive robot at home may be less prominent in rural or suburban areas.

## 7. Study limitations and future work

The goal of this work was to validate the feasibility of using an integrated robot and wearable sensor system to administer guided walking tasks to older adults in out-of-the-lab settings. While the results validated feasibility, this study had several limitations.

First, as the robot we used is a wheeled mobile robot, it suffers from locomotion limitations. For example, it cannot navigate stairs and its navigation performance deteriorates when moving on uneven terrains. In our tests, the electronic walkway that was used as the reference system for performance validation posed locomotion challenges for the robot. While the robot could navigate on and off the walkway, this caused small vibrations to the on-board RGB-D sensors that negatively affected the body tracking performance during brief time periods.

Second, as the robot uses RGB-D sensors for SLAM, it is sensitive to light conditions of the environment. While we found that the robot was generally robust to the indoor lighting in different weather conditions such as rainy or sunny days, in one instance the direct sunlight coming through a sky-window in the common area of the community center where tests were carried out interfered with the Kinect sensor’s FOV, so that the robot could not localize itself correctly. This limitation can be mitigated in our future work by adding other sensors (such as Lidars) that are not sensitive to lighting conditions, at the cost of more expensive sensors on the robot.

Furthermore, due to the exploratory nature of this study, we enrolled a relatively small sample of older adults. Thus, the results we obtained might not be representative of the general population of community-dwelling older adults. The limited sample size also prevented us from compensating for additional confounding factors (e.g., race, ethnicity, number of medications, etc.) which are known to affect gait and balance. However, a sample size of 22, with an alpha level of 0.05, power of 0.8, with three independent predictors in our multiple regression models, allowed us to obtain a moderate effect size of 0.65. Despite the limitations, our work allowed us to validate a novel integrated system that can potentially be used outside the confines of a laboratory situation. In addition, the novel system was able to accurately collect gait and MoS data that were associated with standardized clinical tests of cognition (MoCA) and physical performance (SPPB). The combined results from the clinical tests and integrated novel system highlight the importance of including gait in routine clinical assessment of physical performance in older adults. In addition, the results will be helpful in designing exercise interventions to improve balance, mobility, and strength and potentially reduce falls in older adults.

Future work will include quantifying participants’ performances in the cognitive task, in order to investigate potential mediating effects of task prioritization on the gait patterns measured during the dual-task condition (Fallahtafti et al., 2021).

## 8. Conclusion

In this article, we presented a feasibility study for an integrated mobile robot and wearable sensors system designed to administer guided walking exercises to older adults in out-of-the-lab conditions. The robot-guided study participants to walk on a designated oval path while maintaining a predefined distance from them. During the walking exercises, the robot onboard computer fused data obtained by the robot RGB-D sensor and the insole-embedded sensors to estimate spatiotemporal gait parameters and MoS in real time. The accuracy of the system was assessed against a reference electronic walkway, demonstrating the feasibility of the proposed approach. Associations between gait metrics, physical performance, and cognitive ability were discussed. A subject attitude survey revealed general acceptance of the robotic system by the study participants. Future work will include using the integrated robot and wearable sensors system to assess longitudinal changes in gait and dynamic balance in older adults following a multi-session gait rehabilitation program.

## Footnotes

1
https://wiki.ros.org/dwa_local_planner.
