# Gait monitoring for older adults during guided walking: An integrated assistive robot and wearable sensor approach – ERRATUM

**DOI:** 10.1017/wtc.2022.27

**Published:** 2023-01-06

**Authors:** Qingya Zhao, Zhuo Chen, Corey D. Landis, Ashley Lytle, Ashwini K. Rao, Damiano Zanotto, Yi Guo

A ‘Notes on Contributor’ section containing incorrect information was added to the article. Cambridge University Press & Assessment apologise for the error.
